# Epidemiologic Questionnaire (EPI-Q) – a scalable, app-based health survey linked to electronic health record and genotype data

**DOI:** 10.4178/epih.e2023074

**Published:** 2023-08-08

**Authors:** Maxwell Salvatore, Dylan Clark-Boucher, Lars G. Fritsche, Jacob Ortlieb, Janet Houghtby, Anisa Driscoll, Bryanne Caldwell-Larkins, Jennifer A. Smith, Chad M. Brummett, Sachin Kheterpal, Lynda Lisabeth, Bhramar Mukherjee

**Affiliations:** 1 Department of Biostatistics, University of Michigan, Ann Arbor, MI, USA; 2 Center for Precision Health Data Science, University of Michigan, Ann Arbor, MI, USA; 3 Department of Epidemiology, University of Michigan, Ann Arbor, MI, USA; 4 Rogel Cancer Center, University of Michigan, Ann Arbor, MI, USA; 5 Precision Health, University of Michigan, Ann Arbor, MI, USA; 6 Survey Research Center, Institute for Social Research, Ann Arbor, MI, USA; 7 Anesthesiology, Michigan Medicine, Ann Arbor, MI, USA

**Keywords:** Survey, Electronic health records, Genetics, Epidemiology, Biobank

## Abstract

The Epidemiologic Questionnaire (EPI-Q) was established to collect broad, uniform, self-reported health data to supplement electronic health record (EHR) and genotype information from participants in the University of Michigan (UM) Precision Health cohorts. Recruitment of EPI-Q participants, who were already enrolled in 1 of 3 ongoing UM Precision Health cohorts—the Michigan Genomics Initiative, Mental Health Biobank, and Metabolism, Endocrinology, and Diabetes cohorts—began in March 2020. Of 54,043 retrospective invitations, 5,577 individuals enrolled, representing a 10.3% response rate. Of these, 3,502 (63.7%) were female, and the average age was 56.1 years (standard deviation, 15.4). The baseline survey comprises 11 modules on topics including personal and family health history, lifestyle, and cancer screening and history. Additionally, 11 optional modules cover topics including financial toxicity, occupational exposure, and life meaning. The questions are based on standardized and validated instruments used in other cohorts, and we share resources to expedite development of similar surveys. Data are collected via the MyDataHelps platform, which enables current and future participants to share non-Michigan Medicine EHR data. Recruitment is ongoing. Cohort data are available to those with institutional review board approval; for details, contact the Data Office for Clinical and Translational Research (DataOffice@umich.edu).

## INTRODUCTION

The Epidemiologic Questionnaire (EPI-Q) study was established to collect demographic, physical and social activity, and other health-related data from participants in Precision Health biobank cohorts at the University of Michigan (UM). The earliest of these cohorts, the Michigan Genomics Initiative (MGI) [1], began ongoing recruitment of participants primarily in the perioperative period and has since expanded to include over 90,000 individuals as of September 12, 2022. A newer cohort, Michigan and You – Partnering to Advance Research Together (MY PART), is actively recruiting adults within and beyond Michigan Medicine (MM), primarily via outpatient clinics, patient portals, and emails (with 2,432 participants as of July 7, 2022). All participants in Precision Health cohorts, including MGI and MY PART, provide broad consent. This means that enrollees consent to (1) access to their electronic health records (EHRs), (2) biospecimen genotyping, (3) use and linkage of their data, and (4) recontact for undefined future research. Built primarily on EHR and genotype data, these studies are linked to other data sources, including cancer registry data, vital statistics, neighborhood-level exposures, and prescription claims data (Supplementary Material 1).

**Figure 1.**
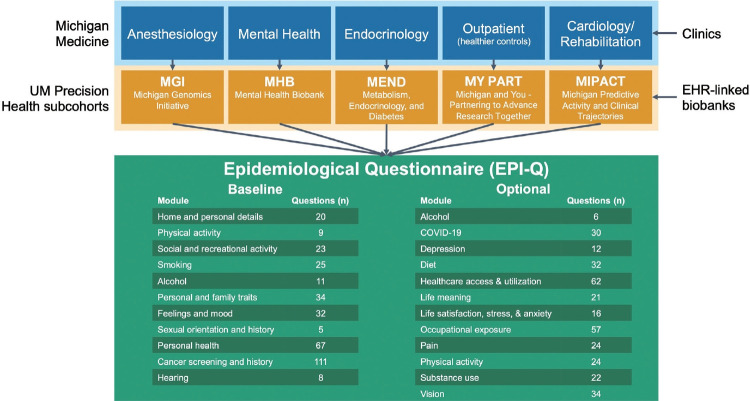
A simplified schematic representation of EPI-Q contents by domain and relationship between Michigan Medicine clinics and related University of Michigan (UM) Precision Health cohorts. EHR, electronic health record; COVID-19, coronavirus disease 2019.

EPI-Q was initially developed for MGI, the largest and longeststanding cohort among the UM Precision Health studies, with the aim of re-engaging individuals (Figure 1). Because MGI recruitment occurs at preoperative or perioperative appointments for surgical and diagnostic procedures requiring anesthesia, the cohort is less healthy overall than the general patient population. This makes it a rich source for studying nearly all disease outcomes [2]. Numerous publications, many related to genetics, have been produced using MGI data [3-5]. Researchers conducting genetic analyses, including those utilizing MGI data, often employ external samples as derivation, validation, or replication cohorts. The UK Biobank [6,7], arguably the most well-known and democratized EHR-linked biobank worldwide, has made its data available to researchers across the globe, making it a popular choice for external samples in genetic analyses. In addition to linking EHR information with genotype data, the UK Biobank has gathered extensive self-reported data across various health-related domains, including behavior and lifestyle. Such data are often incomplete or absent in standard EHR-linked databases (Supplementary Material 2 for a discussion of the limitations of EHR data for research). Thanks to its collection of survey data, the UK Biobank can define phenotypes and adjust for confounders not present in EHR and genotype data.

Considering the standard set by the UK Biobank, the EPI-Q survey was designed to include domains and instruments found in the UK Biobank questionnaire. This not only helps account for potential confounders, but also ensures that the survey items are similar, enabling UM Precision Health cohorts to participate in consortium-wide analyses of survey items and biobank-based meta-analyses. Furthermore, given the numerous UM Precision Health cohorts, the collection of consistent self-reported data across them aids in facilitating multi-cohort analyses. In addition to MGI (originally known as the Anesthesiology Collection Effort) and MY PART, other Precision Health cohorts include the Metabolic, Endocrinology, and Diabetes (MEND), Mental Health Biobank (MHB), and Michigan Predictive Activity and Clinical Trajectory (MIPACT) cohorts. Some of these cohorts were recruited through specific clinics, such as the metabolism, endocrinology, and diabetes clinics for MEND and mental health clinics for MHB (Figure 1), while MIPACT is designed to collect wearable data from MM patients. MY PART is collaborating with partners outside of MM to expand to Detroit (77.9% Black [8]), Flint (56.7% Black [8]), Dearborn (44.9% Arab [9]), and Grand Rapids (15.7% Hispanic [8]). This expansion aims to diversify the cohort and position UM Precision Health as a leading resource for Arab/Middle Eastern/North African health, given the large population residing in Michigan [10]. EPI-Q was developed with the future in mind: recruitment, consent, and survey administration, as well as participant-initiated external EHR sharing protocols, were designed to be safe, secure, scalable, and extendable to cohorts under the UM Precision Health umbrella.

In essence, EPI-Q allows users of UM Precision Health cohort data to better adjust for confounders, compare participants across cohorts, and engage in consortium-wide and external meta-analyses, particularly with the UK Biobank. In addition, data collection on topics including financial toxicity, occupational exposures, and life meaning broadens its appeal to researchers beyond the realms of health and genetics.

## STUDY PARTICIPANTS

Participants are invited to take part in the study via email. Consent and surveying are managed electronically through the MyDapants have the option to enroll and participate either through the MyDataHelps mobile application or the web browser platform on a computer. Once the consent process is complete, participants may fill out the one-time baseline survey. Upon completion, optional modules become available on the participant’s dashboard within the MyDataHelps platform. All survey modules are administered simultaneously (i.e., cross-sectionally).

While the EPI-Q survey data are collected cross-sectionally, the consent and data collection platform allows us to consider new survey modules on timely topics (indeed, an optional coronavirus disease 2019 [COVID-19] module has already been added) as well as potentially longitudinal responses of baseline data. Additionally, the EHR data and other linkable databases are updated on an ongoing basis.

The primary recruitment mechanism targeted adults receiving anesthesiology for surgical or diagnostic procedures (91%), with some participants recruited from mental health (1%) or metabolism, endocrinology, and diabetes (6%) clinics, as well as a wearable data study (2%). Inclusive of the pilot phase (Supplementary Materials 3 and 4), a total of 54,043 invitations were sent to all MGI participants who (1) were alive at the time of EPI-Q recruitment, (2) had an email address registered in the UM Patient Portal, and (3) had a biospecimen on file (not necessarily genotyped yet). Participants were incentivized with an ancestry report upon completion of the baseline survey. Of those invited, 5,577 enrolled, constituting a response rate of 10.3%. Descriptive statistics comparing EPI-Q respondents and non-respondents through June 30, 2022 are summarized in Table 1 (with a comparison of EHR-derived vs. EPI-Q self-report data presented in Supplementary Material 5).

Based on data extracted from EHRs (Supplementary Material 6), the respondents were generally slightly younger, with a mean age of 56.1 compared to 57.5 years (p= 0.03). They were more likely to be female (64 vs. 56%; p< 0.01), White (92 vs. 90%; p< 0.01), and married (57 vs. 54%; p< 0.01). They were also more likely to have consumed alcohol (80 vs. 73%; p< 0.01) and to have never smoked (62 vs. 55%; p< 0.01). Multivariable logistic regression models corroborated the findings that the response likelihood was higher among female participants and married individuals, and lower among non-Hispanic Blacks and both current and former smokers (Supplementary Materials 7 and 8). The respondents were less likely to have qualified through recruitment into MGI (84 vs. 91%; p< 0.01). This is probably because the MGI recruitment process has been ongoing for longer than the other cohorts. In other words, participation rates have tended to increase as the time since enrollment in a qualifying cohort (MGI, MEND, MHB, MIPACT) decreased.

**Figure 2.**
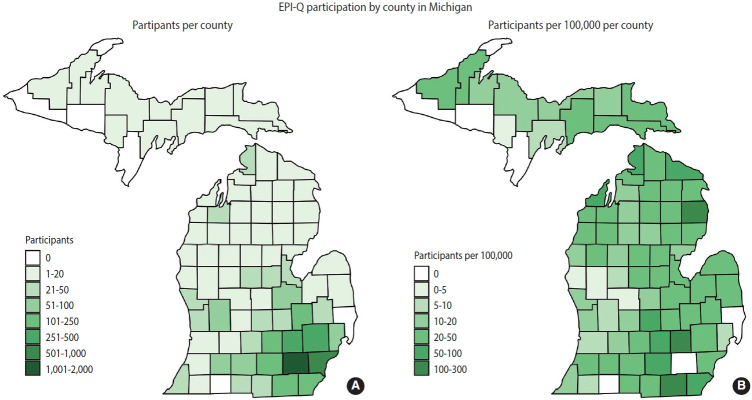
A county-level map representing Epidemiologic Questionnaire (EPI-Q) participants (n=5,498) as raw counts (A) and per 100,000 of total county population (B) based on their residential address as reported in their Michigan Medicine patient portal. EPI-Q participants who do not have available residential addresses are excluded. (A) Out of 5,498 participants. Participants without county information (n=66) or who live out of state (n= 366) not shown. (B) County population data from 2020 Census Demographics and Housing Survey.

Participants in EPI-Q, who are drawn from the UM Precision Health cohorts, predominantly represent the MM catchment area. This area encompasses the central, lower peninsula of Michigan, and its population is more predominantly White than the overall population of Michigan. Figure 2 provides a county-level map showing the origins of EPI-Q participants, with a relative distribution detailed in Supplementary Material 9. Notably, some participants, primarily from neighboring states, were eligible for MGI because they traveled to MM for specialized surgical procedures that required anesthesia. Table 2 presents a comparison of the state of Michigan, MM, and UM Precision Health cohorts regarding size, age, sex, and race/ethnicity. A more detailed comparison between UM Precision Health and EPI-Q participants can be found in Supplementary Material 10. Future participants from UM Precision Health cohorts, both within and beyond MM, will be invited to participate, as shown in Supplementary Material 11.

### Ethics statement

This study received ethical and regulatory approval from the Institutional Review Boards (IRBs) of the University of Michigan Medical School (IRBMED), under IRB No. HUM00155782.

## MEASUREMENTS

The EPI-Q questionnaire was primarily modeled after the tool used by the UK Biobank, with the aim of enabling UM Precision Health cohorts to participate in meta-analyses. The UK Biobank questionnaire collects extensive data on socio-demographic factors, family and early life history, psychosocial aspects, lifestyle, medical history, and cognitive function. It is administered to participants in person.

We began by reviewing the UK Biobank questionnaire regarding the relevance and applicability of its questions to a United States context. The questions were then categorized into broad domain-based modules such as alcohol use, sexual history, and feelings and mood. Following this initial review, we compared these questions with those used in other United States-based biobanks and UM cohort studies. These included the National Institutes of Health All of Us Research Program [11], the Veterans Administration Million Veteran Program [12], and the UM Genes for Good initiative [13]. These foundational resources were selected due to their potential for (genome-wide association study [GWAS]) metaanalyses, with a particular focus on the UK Biobank. The resulting structure comprised 11 baseline modules and 11 optional modules, with a COVID-19 module added later (Figure 1). Supplementary Material 12 highlights the overlap between questions in EPI-Q and the UK Biobank. As detailed in Supplementary Material 13, several modules encompass multiple domains as defined by the study team.

**Figure 3.**
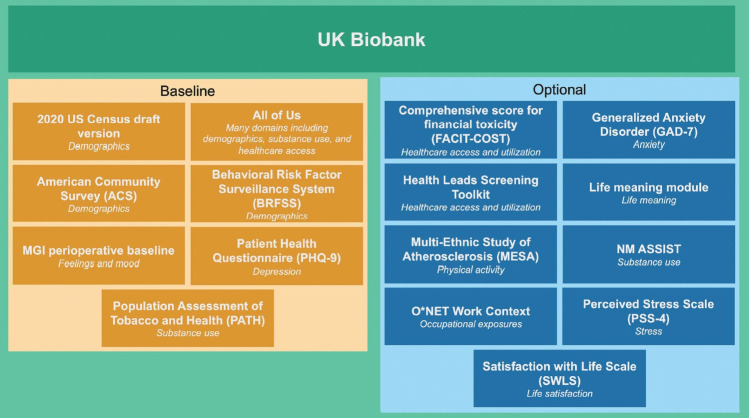
A diagram summarizing different surveys that were consulted in the development of the Epidemiological Questionnaire along with their respective domains (in italics). MGI, Michigan Genomics Initiative; MM, Michigan Medicine.

Experts from various departments at UM, including Anesthesiology, Biostatistics, Environmental Health Science, Epidemiology, Health Behavior and Health Education, Occupational Health, Oncology, and Psychiatry, recommended domain-specific survey instruments and reviewed the resulting portions of the survey. The UM Survey Research Center reviewed the drafts to assess participant burden, to ensure consistency with UM panel surveys and widely used national surveys, and to verify the appropriateness of wording and responses in line with evidence-based survey practices (Figure 3). A comprehensive table listing all questions and their corresponding sources can be accessed online (https://www.doi.org/10.17605/OSF.IO/PBY9J) [14].

The EPI-Q questionnaire consists of 22 modules, divided into baseline and optional surveys. The baseline survey includes 11 modules: alcohol use, cancer history and screening, family, feelings and mood, hearing, home and personal details, personal health, physical activity, sexual orientation and history, smoking, and social and recreational activity. The incentive for baseline survey completion is a UM-generated ancestry report, which uses genotyped biospecimens collected from the participant’s enrollment in a UM Precision Health cohort that qualifies for EPI-Q.

The optional surveys also include 11 modules: depression, diet and eating habits, healthcare access and utilization, life meaning, life satisfaction, anxiety and stress, occupational exposures, pain, physical activity, substance use, and vision. These modules were selected to reduce the participant burden for baseline completion and incentive receipt, while still addressing areas of interest to the research team and collaborators. They either elaborate on topics already covered in the baseline survey (e.g., alcohol use) or collect new information (e.g., healthcare access and utilization). Following completion of the pilot phase, an additional optional COVID-19 module received IRB approval. This module seeks information about diagnoses, exposure and symptom history, and vaccination history and hesitancy.

In Supplementary Material 14, we highlight 3 survey instruments that are less commonly used or represent emerging areas of research: occupational exposure, financial toxicity, and life meaning.

### Completion rates

Completion rates, defined as the proportion of enrollees who completed any part of a given survey to which they had access, were high among the 5,498 participants for the incentivized baseline modules. These rates ranged from 80.2% for the personal health module to 94.4% for the personal and family attributes module. An impressive 79.9% (n= 4,393) of participants completed all baseline modules. Completion rates were lower for the non-incentivized optional modules, although they remained relatively high. These rates ranged from 62.7% for life satisfaction to 75.8% for vision. The optional occupational exposure module had a noticeably lower completion rate of 44.2%. Among those who completed any of the optional modules, 38.0% (n= 1,841) finished all 11 of the original optional modules (excluding the COVID-19 module, which was added later). The completion rates by module are summarized in Supplementary Material 15.

### Survey length (time to complete)

Survey lengths for each module, as well as the baseline and optional surveys, were assessed in the original sample of 601 participants, following the distribution of the initial 5,000 invitations. After removing outliers (those outside of the interquartile range; IQR ± 1.5 × IQR), the average completion time for the baseline survey was determined to be 20.8 minutes (95% confidence interval [CI], 11.5 to 30.0), with an average of 163.3 questions answered across 10.8 of the 11 modules. The optional survey had an average completion time of 19.1 minutes (95% CI, 5.1 to 33.1). The average time to complete each module ranged from 0.7 minutes for the baseline sexual orientation and history module to 5.2 minutes for the optional occupational exposure module. The average survey lengths for each module are summarized in Supplementary Material 16.

## KEY FINDINGS

### Concordance between Epidemiologic Questionnaire and electronic health record for sex and cancer history variables

Several variables are recorded in both the EHR and EPI-Q. In this study, we examined the concordance of sex as reported in these 2 sources: self-reported sex at birth via EPI-Q and archived sex as documented in the EHR. We assessed concordance using the Cohen kappa (κ), a measure of the proportion of cases in agreement while accounting for the number of agreements expected to occur by chance. Despite some discrepancies, we generally noted extremely high consistency, with κ= 0.986 (self-report vs. EHRrecorded; Supplementary Material 17).

Unlike sex, a substantial disagreement was observed regarding cancer history (Supplementary Materials 18 and 19). Among the 1,850 respondents who reported a history of cancer, 96.7% (n= 1,789) had a cancer diagnosis documented in their health records (see qualifying phecodes in Supplementary Material 20). This discrepancy could be due to several factors: (1) certain types of cancer may not have been included in our EHR-based classification, (2) individuals may have inaccurately reported their cancer history, such as misinterpreting non-positive cancer screening results or including precancerous lesions, or (3) individuals may have received a cancer diagnosis from another healthcare provider, and thus, the diagnosis was not recorded in their MM EHR. Among individuals with a history of cancer as documented in their EHR, self-reported cancer history varied based on the type of cancer, ranging from 100% (e.g., myeloid leukemia, chronic) to 17% (neurofibromatosis) (Supplementary Material 20). More nuance is explored in Supplementary Material 18, including the distribution of time since cancer diagnosis (Supplementary Material 21), self-report by time since cancer diagnosis (Supplementary Material 22), and exploratory models for cancer self-report (Supplementary Material 23).

### Differences in occupational exposures, financial toxicity, and life meaning by self-reported history of cancer

The unique data fields of EPI-Q have the potential to generate novel research questions across scientific fields. Initially, we analyzed responses to questions about occupational exposures based on self-reported cancer history (Supplementary Material 24). We observed only 2 statistically significant differences: individuals without a self-reported history of cancer were more likely to report (1) exposure to a cramped workspace and (2) walking or running as part of their job.

Second, we compared responses to questions regarding financial toxicity by self-reported history of cancer (Supplementary Material 25). Unlike occupational exposure, we observed many statistically significant differences in the responses. However, a self-reported history of cancer did not consistently correlate with greater financial toxicity. Importantly, questions about financial toxicity were posed only to individuals who reported receiving “treatment for a new or ongoing illness or condition in the past 7 days.” Therefore, many people with a self-reported cancer history were likely responding to these questions in relation to a noncancer illness or condition. Furthermore, we are only presenting the mean differences between those with and without a self-reported history of cancer, without considering potential confounding factors such as age, employment, or income.

Third, we analyzed responses to questions about life meaning based on self-reported cancer history (Supplementary Material 26). The Comprehensive Measure of Meaning instrument, which we refer to as “life meaning” in this context, is divided into 3 domains: coherence (6 questions), significance (6 questions), and direction (9 questions). Each question was rated on a 7-point Likert scale, with 1 indicating strong disagreement and 7 indicating strong agreement. For each domain, we calculated a simple average of the non-missing responses, both by domain and overall for each individual. In each domain, as well as overall, individuals with a self-reported history of cancer reported significantly higher levels of life meaning. It is important to reiterate that we are reporting mean differences in life meaning scores based on self-reported cancer history, without considering potential confounding factors such as age, sex, education, or income. The distribution of average scores, both overall and for each domain, is depicted in Supplementary Material 27.

### Life meaning: genome-wide association study

We conducted a proof-of-concept GWAS focused on life meaning. The study of life meaning, along with other psychosocial measures such as life purpose, life satisfaction, and happiness, has been increasing [15,16]. This includes studies that employ genetic analyses [17,18]. Our sample consisted of 2,433 participants, primarily of European ancestry (as inferred from available genotype data), who answered at least 1 question in the life meaning module. We conducted a separate GWAS for each domain of life meaning and for overall life meaning (Supplementary Material 28) using ENCORE, a web-based analysis tool used for GWAS at UM. All GWAS analyses were executed with an inverse normalized outcome using a fast linear mixed model with kinship adjustment (SAIGE [19]). All models were adjusted for age at survey, sex, qualifying study, genotyping batch, and the first 10 principal components of the genotype data. We considered variants with a minor allele frequency greater than 0.1% and a minor allele count exceeding 20.

Notably, these results are solely for demonstrative purposes. We observed no clear association signal that deviated significantly from random findings. Future research in this field should carefully consider the operationalization of the questions in the life meaning module. When used alongside MGI, the EPI-Q study provides a gateway to a wide array of research questions that may not be answerable through other means, particularly questions that involve creative applications of genetic data.

## STRENGTHS AND WEAKNESSES

This survey has several strengths. First, the survey benefits from construction on an existing, large, and institutionally supported cohort base. Participants in the EPI-Q survey are individuals who are interested and actively involved in research, drawn from a growing and sustainable pool of cohorts. Second, due to its administration electronically in the form of a mobile app, the survey is scalable. It can be administered to tens of thousands of current and future participants in a relatively short period, and thanks to remote electronic consent, it can reach a geographically diverse area. Furthermore, the setup and consent processes are flexible and can accommodate future longitudinal data capture through the existing data collection infrastructure. Third, this survey incorporates established survey instruments that include questions from the UK Biobank, the National Institutes of Health *All of Us* initiative [11], the Million Veteran Program [12], and the 9-item Patient Health Questionnaire (PHQ-9) [20]. This, coupled with the numerous potential data linkages, positions the EPI-Q resource favorably for meta-analyses and validation studies. Fourth, Michigan is home to one of the largest Middle Eastern and North African populations in the United States. Our initiative is uniquely positioned to establish a large health-related database on the Arab/Middle Eastern/North African community, a group for which data are currently lacking for health research [21]. Fifth, this survey addresses a data gap and is crucial for enhancing our holistic understanding of health and well-being. This gap exists due to the imperfections and inaccuracies in our EHR data, bearing in mind that EHRs are not designed for research purposes. Additionally, the MyDataHelps platform enables users of non-MM healthcare systems to share their primary care EHR record with us, thereby minimizing known issues associated with using academic medical center-based EHR data for research [2].

The EPI-Q study also has several weaknesses. First, the current participant base lacks diversity, being predominantly White. This exceeds the proportion of White individuals both in the state and within MM. Historically, health research, especially genomic research, has been disproportionately focused on White/European males [22]. Our team, along with Precision Health, is actively working to increase diversity so that both the cohort and the research outcomes can better serve the people of Michigan and beyond. Second, in engaging with a broad group of diverse researchers, decisions had to be made regarding the nature of the survey questions included. We recognize that our broad epidemiologic questionnaire may not delve into preferred instruments in sufficient detail for many well-defined, domain-specific research questions. Third, the various study populations and recruitment mechanisms used to acquire the participant cohorts for EPI-Q present analytical challenges in obtaining internally consistent and externally valid results (Table 1 and Figure 1, Supplementary Material 8). Fourth, our cross-tables of EHR-based and self-reported cancer histories reveal a significant number of individuals who have qualifying cancer diagnoses in their EHR but did not self-report this information. This discrepancy could stem from the qualifying cancer PheWAS codes (phecodes) used (listed in Supplementary Material 20) being too broad, meaning that some qualifying cancer phecodes may not have been recognized as a cancer diagnosis by the patient. We have reported the proportion of individuals who self-report cancer by qualifying cancer phecode (Supplementary Material 20), but 94.5% of individuals with an EHR-defined history of cancer have multiple qualifying cancer phecodes on their EHR, complicating this area for future work. Fifth, while the financial toxicity module is based on the validated FACIT-COST instrument, it was originally validated in cancer patients [23,24] and was modified to consider any recent chronic condition for use in EPI-Q. However, since its development and EPI-Q use, the instrument has been validated in patients with diabetes [25] and in those with chronic conditions [26].

## DATA ACCESSIBILITY

The study-related materials, which include copies of the baseline, optional, and feedback surveys annotated with sources as well as community engagement studio slides, can be found at https://www.doi.org/10.17605/OSF.IO/PBY9J.

The data are available for researchers worldwide, provided that they are associated with a UM-affiliated researcher and have the necessary regulatory approvals to access the data. More information about MGI can be found here (https://precisionhealth.umich.edu/our-research/michigangenomics/), EPI-Q here (https://sph.umich.edu/precision-health-data-science/epi-q/index.html), and UM Precision Health here (https://precisionhealth.umich.edu/). If you are interested in accessing the data, please reach out to Bhramar Mukherjee (bhramar@umich.edu).

## Figures and Tables

**Table 1.** Comparison of EHR-based characteristics among those who did and did not respond to the Epidemiological Questionnaire (EPI-Q)^1^

Characteristics	n	Approached for EPI-Q		p-value^2^
		Non-respondent (n=48,436)	Respondent (n=5,498)	
Age (yr)	53,934			<0.01
Mean±SD	53,933	57.5±16.2	56.1±15.4	<0.01
18-35		5,630 (11.6)	638 (11.6)	
35-50		9,559 (19.7)	1,237 (22.5)	
50-65		15,185 (31.4)	1,743 (31.7)	
65-80		14,827 (30.6)	1,709 (31.1)	
80-100		3,234 (6.7)	171 (3.1)	
Female	53,934	27,318 (56.4)	3,502 (63.7)	<0.01
Race	53,383			<0.01
White		43,118 (90.0)	5,014 (91.9)	
Asian		966 (2.0)	134 (2.5)	
Black		2,635 (5.5)	173 (3.2)	
Other		1,210 (2.5)	133 (2.4)	
Ethnicity	52,353			0.07
Hispanic		45,968 (97.8)	5,186 (97.4)	
Non-Hispanic		1,058 (2.2)	141 (2.6)	
Race/Ethnicity	52,206			<0.01
NHW		41,572 (88.7)	4,802 (90.3)	
NHB		2,558 (5.5)	164 (3.1)	
Other		2,759 (5.9)	351 (6.6)	
BMI	53,854	30 (7.3)	30 (7.4)	0.17
Married	45,850	25,918 (62.9)	3,072 (66.2)	<0.01
Alcohol consumption (ever)^3^	51,203	33,545 (72.9)	4,146 (80.0)	<0.01
Smoking status	53,839			<0.01
Never		26,641 (55.1)	3,402 (62.3)	
Former		14,312 (29.6)	1,502 (27.5)	
Current		7,422 (15.3)	560 (10.2)	
NDI, mean±SD^4^	48,402	3.01±1.01	3.09±1.01	<0.01
Qualifying study^5^	36,089			<0.01
MGI		29,918 (91.2)	2,768 (84.0)	
MEND		1,941 (5.9)	200 (6.1)	
MIPACT		507 (1.5)	256 (7.8)	
MHB		427 (1.3)	72 (2.2)	

Values are presented as number (%). EHR, electronic health record; SD, standard deviation; NHW, non-Hispanic White; NHB, non-Hispanic Black; NDI, neighborhood disadvantage index; BMI, body mass index; MGI, Michigan Genomics Initiative; MEND, Metabolic, Endocrinology, and Diabetes; MHB, Mental Health Biobank; MIPACT, Michigan Predictive Activity and Clinical Trajectories.

1 This table is limited to those who were invited to participate and who are in the phenome file; A total of 48,546 people were invited, and 5,577 enrolled.

2 Calculated via the Welch two-sample t-test for numeric variables and the Pearson chi-square test for categorical variables.

3 Alcohol consumption is captured through Clarity Social History in the electronic health record.

4 The NDI, which is operationalized as quartiles, is the average of the proportions of (i) female-headed families with children, (ii) households using public assistance income, (iii) people with income below the poverty level in the last 12 months, and (iv) the population (age 16 years and older) unemployed at the census tract level; It is based on the participant’s residential address as reported in the Michigan Medicine patient portal.

5 Qualifying study identified by genotype sample; Missingness in this variable is due to unprocessed biospecimen.

**Table 2.** Comparison of Michigan, Michigan Medicine, and UM Precision Health cohorts by size, age, sex, and race/ethnicity

Variables		Precision Health subcohorts					Combined Precision Health cohort	Michigan Medicine^1^	State of Michigan^2^
		MGI-ACE	MEND	MHB	MIPACT	MY PART			
Total (n)		73,178	4,172	2,362	7,619	2,623	90,076	4,369,283	9,970,000
Age (yr)									
	Range	18-106	20-96	20-96	19-97	18-92	15-106	0-91+	-
	Mean	60	58.2	43.6	50.1	40.3	57.6	-	-
	Median	62	60.6	39.9	50.8	37.7	59.7	-	39.8
Sex									
	Male	33,966 (46.4)	2,000 (47.9)	855 (36.2)	3,431 (45.0)	733 (27.9)	40,910 (45.4)	2,003,463 (45.9)	5,060,000 (50.8)
	Female	39,210 (53.6)	2,172 (52.1)	1,506 (63.8)	4,188 (55.0)	1,890 (72.1)	49,162 (54.6)	2,315,128 (53.0)	4,910,000 (49.2)
	Other	2 (0.0)	0 (0.0)	1 (0.0)	0 (0.0)	0 (0.0)	4 (0.0)	8,983 (0.2)	-
	Unknown	0 (0.0)	0 (0.0)	0 (0.0)	0 (0.0)	0 (0.0)	0 (0.0)	41,699 (1.0)	-
Race/Ethnicity									
	White, non-Hispanic	64,822 (88.6)	3,493 (83.7)	2,019 (85.5)	3,777 (49.6)	1,663 (63.4)	75,788 (84.1)	2,580,565 (59.1)	7,430,000 (74.5)
	Black, non-Hispanic	3,727 (5.1)	399 (9.6)	130 (5.5)	1,284 (16.9)	200 (7.6)	5,768 (6.4)	295,581 (6.8)	1,340,000 (13.5)
	Other, non-Hispanic	3,153 (4.3)	165 (4.0)	133 (5.6)	1,687 (22.1)	606 (23.1)	5,823 (6.5)	1,426,360 (32.6)	678,000 (6.8)
	Hispanic	1,476 (2.0)	116 (2.8)	80 (3.4)	871 (11.4)	154 (5.9)	2,698 (3.0)	66,777 (1.5)	521,000 (5.2)

Values are presented as number (%). UM, University of Michigan; MGI, Michigan Genomics Initiative; ACE, Anesthesiology Collection Effort; MEND, Metabolism, Endocrinology & Diabetes; MHB, Mental Health Biobank; MIPACT, Michigan Predictive Activity and Clinical Trajectories study; MY PART, Michigan and You – Partnering to Advance Research Together.

1 Data on patients who are not recorded as deceased.

2 Data according to 2020 American Community Survey 5-year estimates.

## SUPPLEMENTARY MATERIALS

Supplementary Material 1. Linkable data on University of Michigan Precision Health cohort participants (including those in the Michigan Genomics Initiative) who complete the Epidemiological Questionnaire.

Supplementary Material 2 Brief comment on limitations to use of electronic health record data for research

Supplementary Material 3 Description of the pilot phase

Supplementary Material 4 Summary of responses to selected questions on the feedback survey administered to the 601 pilot respondents. 556 individuals completed any part of the feedback survey.

Supplementary Material 5 Comparison of EHR-derived and self-reported characteristics among 5,498 EPI-Q participants

Supplementary Material 6 Flowchart describing respondents and those with EHR data available for nonresponse analyses. Dashed lines represent individuals that due to missing data were excluded at the corresponding step.

Supplementary Material 7 Factors associated with response to EPI-Q invitation

Supplementary Material 8 Multivariable logistic regression models of response to survey vs nonresponse

Supplementary Material 9 Proportion of cohort population by county in Michigan

Supplementary Material 10 Comparison of UM Precision Health participants and EPI-Q participants based on data available in the electronic health record.

Supplementary Material 11 Schematic describing the recruitment process from the Michigan Genomics Initiative into the Epidemiological Questionnaire.

Supplementary Material 12 The number of questions in EPI-Q by baseline and optional module and the proportion of questions that also appear in the UK Biobank questionnaire. The gray bars represent the number of questions in the EPI-Q module while the orange portion of each bar represents the proportion of questions that overlap with the UK Biobank.

Supplementary Material 13 Modules, their domains, and number of questions in baseline and optional EPI-Q surveys

Supplementary Material 14 Highlighting EPI-Q survey instruments that are less common or represent upcoming areas of research

Supplementary Material 15 Proportion of participants who completed EPI-Q by baseline and optional modules. Baseline modules were required for participants to receive the incentive; there was no incentive for completing optional modules.

Supplementary Material 16 Average time-to-complete (95% confidence interval; in minutes) baseline and optional modules in the Epidemiological Questionnaire (EPI-Q). Based on pilot data from 601 respondents from 5,000 initial invitations after removing individual responses with completion times that qualify as outliers (IQR±1.5×IQR).

Supplementary Material 17 Comparison of self-reported and EHR-recorded concordance

Supplementary Material 18 Comments regarding discrepancy between EHR-defined and self-reported history of cancer

Supplementary Material 19 Concordance between self-reported history of cancer and EHR-recorded diagnosis of cancer

Supplementary Material 20 Occurrence of qualifying cancer International Classification of Disease (ICD)-derived traits (phecodes as first described by Denny and colleagues (26,27)) and the proportion of individuals who self-reported history of cancer

Supplementary Material 21 Time since most recent initial cancer diagnosis by individual.

Supplementary Material 22 Proportion of EHR-defined cancer patients who self-reported history of cancer via EPI-Q by decile of time since most recent initial cancer diagnosis.

Supplementary Material 23 Logistic regression models for self-reported history of cancer under three sets of covariates.

Supplementary Material 24 Responses to occupational exposure questions by self-reported history of cancer.

Supplementary Material 25 Responses to financial toxicity questions by self-reported history of cancer.

Supplementary Material 26 Comparison of average life meaning scores by domain and overall by self-reported history of cancer

Supplementary Material 27 Distribution of average life meaning scores overall and by domain among 4,125 participants who completed any part of the life meaning module.

Supplementary Material 28 Manhattan plot summarizing p-values from life meaning GWAS of 2,433 EPI-Q participants who completed at least one question in the corresponding module.

Supplementary References
