# Supplementary material for: Epidemiologic Questionnaire (EPI-Q) – a scalable, app-based health survey linked to electronic health record and genotype data
Source: Epidemiol Health. 2023 Aug 8;45:e2023074. doi: 10.4178/epih.e2023074 (PMC10867525; doi:10.4178/epih.e2023074)
Supplement: Supplementary Material 1. — Linkable data on University of Michigan Precision Health cohort participants (including those in the Michigan Genomics Initiative) who complete the Epidemiological Questionnaire. [file epih-45-e2023074-Supplementary-1.docx]

*Supplementary material to…*

Cohort Profile: Epidemiologic Questionnaire (EPI-Q) – an app-based, scalable health survey linked with electronic health record and genotype data

**Supplementary Material 1**. Linkable data on University of Michigan Precision Health cohort participants (including those in the Michigan Genomics Initiative) who complete the Epidemiological Questionnaire.
