# Supplementary material for: Epidemiologic Questionnaire (EPI-Q) – a scalable, app-based health survey linked to electronic health record and genotype data
Source: Epidemiol Health. 2023 Aug 8;45:e2023074. doi: 10.4178/epih.e2023074 (PMC10867525; doi:10.4178/epih.e2023074)
Supplement: Supplementary Material 2 — Brief comment on limitations to use of electronic health record data for research [file epih-45-e2023074-Supplementary-2.docx]

**Supplementary Material 2. Brief comment on limitations to use of electronic health record data for research**

While there are numerous benefits to the use of EHR data for secondary research -including reduction of study costs, expansive and systematically collected longitudinal data, and hypothesis generation through agnostic association analyses - there are significant concerns. Primarily, EHR data is not collected with research use in mind; they are driven by insurance, billing, and follow-up care considerations and incentives (1). This is exemplified by differences in EHR data coding and recording practices across physicians, departments, and health systems (2–4). Research shows that there are other nuances of EHR data that result in selection and information biases (e.g., misclassification) (5). These data quality issues remain without a consensus as to the quality of EHR data (6). Efforts to create methods to assess and account for these issues are underway, including improvement in the extraction of unstructured EHR data (e.g., clinical notes) via natural language processing (7,8). However, it is unlikely that these developing methods will resolve all data quality issues for research use. Other parallel efforts to gather additional, standard data (9) to augment and complement EHR data are necessary for rigorous health research; indeed, many prominent EHR-linked biobanks collect survey data alongside their EHR and biospecimen data (see Table 1 from Beesley and colleagues (10)) (11–13).
