# Supplementary material for: Epidemiologic Questionnaire (EPI-Q) – a scalable, app-based health survey linked to electronic health record and genotype data
Source: Epidemiol Health. 2023 Aug 8;45:e2023074. doi: 10.4178/epih.e2023074 (PMC10867525; doi:10.4178/epih.e2023074)
Supplement: Supplementary Material 3 — Description of the pilot phase [file epih-45-e2023074-Supplementary-3.docx]

**Supplementary Material 3. Description of the pilot phase**

The pilot phase of the study represents a random sample of 5,000 participants stratified by sex, age, and race/ethnicity. An initial invitation was sent to 300 of these participants to ensure there were no logistical or technical errors before inviting the full 5,000. During this phase, up to 3 invitation emails (one week apart) were sent and followed by a phone call. After this, the follow-up phone call was removed from the recruitment approach (i.e., eligible individuals only received up to 3 invitation emails). The pilot phase was completed after sending the initial 5,000 invitations. Of these invitations, 601 enrolled.

During the pilot phase, invitations were sent to previously enrolled MGI participants who were alive at recruitment, had an email address in the UM Patient Portal, and had a genotyped biospecimen on file. Pilot participants were eligible to choose to receive either a $10 gift card or a private ancestry report (conducted by the University of Michigan Center for Statistical Genetics using genotype data from participants’ MGI enrollment) for completion of the baseline modules. Based on participants’ overwhelming preference for an ancestry report seen in the pilot phase, future participants will only be able to receive an ancestry report.

Pilot feedback survey

We administered a feedback survey to 601 participants from the initial 5,000 invitations, of whom 556 responded (92.5% response rate). The pilot feedback survey was administered to get user feedback on survey length, ease of understanding instructions and questions, and comfortability. Feedback was overwhelmingly positive. Respondents felt the instructions for completing the survey were “very clear” (90.3%, n = 502) and that the questions were “very easy to understand” (89.0%, n = 495). Moreover, participants felt comfortable with the questions that were asked (95.5%, n = 531) and that the survey was not too long (84.9%, n = 472). Finally, a substantial majority of respondents (84.4%, n = 469) were willing to be contacted by the study team for additional, in-depth feedback. A summary of responses to the feedback survey is in **Supplementary Material 4**.

Community engagement studio

Among individuals who expressed willingness to be contacted for additional feedback in the feedback survey, we held a community engagement studio (CES; i.e., focus group) with five participants on February 19, 2021, to obtain feedback on six concepts: recruitment and enrollment, experience using survey software (MyDataHelps), incentive structure, ancestry report, knowledge of eligibility, and return of information. The CES was facilitated by the Michigan Institute for Clinical Health Research (MICHR) at UM.

Regarding recruitment and enrollment, CES participants did not express challenges with enrolling but felt that the invitation email was too long, needed to stress the importance of participation, and needed to address concerns about the monetization of data. CES Participants suggested recruiting families (as opposed to individual patients) and participants from other medical systems. They positively commented on the survey software, MyDataHelps, and web browser, and did not encounter issues when using it.

During the pilot phase, participants were given the option of selecting a $10 gift card or receiving an ancestry report from the biospecimen they previously provided through participation in a qualifying study. CES participants expressed some confusion saying they were unaware that they had an option. Others expressed concern that the ancestry report meant their data was being sold or shared. Participants who selected the ancestry report were unsure of when their report was available and how to access their report.

Because individuals must be a part of a UM Precision Health study to be eligible (e.g., MGI) and enrollment into that study may have been years prior, CES participants were asked whether they were aware they were enrolled. Generally, participants remembered they had enrolled and thought this was adequately communicated via EPI-Q recruitment materials. Some did not recall enrolling in a qualifying study.

In summary, the participant studio was an engaged and lively discussion of what people like about EPI-Q. Looking at their lifestyle, diet, and social factors and not just genetics was very appealing and made them feel that EPI-Q views participants as a “whole.” CES participants felt EPI-Q communications could be made more uniform (specifically the inclusion of Precision Health logo to align with the quarterly Precision Health Newsletter).

An anonymous, prompted comment form was administered to participant studio participants. Participants were very supportive saying the “project seems to be worthwhile,” that EPI-Q is “very well organized” and that “participation was easy,” while stressing the importance of providing “a clearer picture of the project […] at the outset” and communicating “the importance of participating in this type of research.”
