# Supplementary material for: Epidemiologic Questionnaire (EPI-Q) – a scalable, app-based health survey linked to electronic health record and genotype data
Source: Epidemiol Health. 2023 Aug 8;45:e2023074. doi: 10.4178/epih.e2023074 (PMC10867525; doi:10.4178/epih.e2023074)
Supplement: Supplementary Material 4 — Summary of responses to selected questions on the feedback survey administered to the 601 pilot respondents. 556 individuals completed any part of the feedback survey. [file epih-45-e2023074-Supplementary-4.docx]

| **Supplementary Material 4**. Summary of responses to selected questions on the feedback survey administered to the 601 pilot respondents. 556 individuals completed any part of the feedback survey. | | |
| --- | --- | --- |
|  | **N** | **%** |
| **Are the instructions for completing the survey clear?** |  |  |
| Yes - very clear | 502 | 90.3 |
| Yes - somewhat clear | 51 | 9.2 |
| No - somewhat unclear | 1 | 0.2 |
| No - very unclear | 0 | 0.0 |
| *Missing* | 2 | 0.4 |
| **Are the questions easy to understand?** |  |  |
| Yes - very easy to understand | 495 | 89.0 |
| Yes - somewhat easy to understand | 61 | 11.0 |
| No - somewhat difficult to understand | 0 | 0.0 |
| No - very difficult to understand | 0 | 0.0 |
| *Missing* | 0 | 0.0 |
| **Are there questions that made you feel uncomfortable?** |  |  |
| No - no question made me feel uncomfortable | 400 | 71.9 |
| No - a couple questions made me feel uncomfortable | 131 | 23.6 |
| Yes - several questions made me feel uncomfortable | 21 | 3.8 |
| Yes - a lot of questions made me feel uncomfortable | 3 | 0.5 |
| *Missing* | 1 | 0.2 |
| **Did you feel that the survey was too long?** |  |  |
| No - survey was not too long | 472 | 84.9 |
| Unsure | 60 | 10.8 |
| Yes - survey was too long | 21 | 3.8 |
| *Missing* | 3 | 0.5 |
| **Can the study team contact you for additional feedback?** |  |  |
| Yes, feel free to contact me | 469 | 84.4 |
| No, please do not contact me | 73 | 13.1 |
| *Missing* | 14 | 2.5 |
