# Supplementary material for: Epidemiologic Questionnaire (EPI-Q) – a scalable, app-based health survey linked to electronic health record and genotype data
Source: Epidemiol Health. 2023 Aug 8;45:e2023074. doi: 10.4178/epih.e2023074 (PMC10867525; doi:10.4178/epih.e2023074)
Supplement: Supplementary Material 5 — Comparison of EHR-derived and self-reported characteristics among 5,498 EPI-Q participants [file epih-45-e2023074-Supplementary-5.docx]

| **Supplementary Material 5**. Comparison of EHR-derived and self-reported characteristics among 5,498 EPI-Q participants | | |
| --- | --- | --- |
|  | **EHR** | **Self-report** |
| Age (mean (standard deviation)) | 56.1 (15.4) | 56.3 (14.9) |
| Age category |  |  |
| [18, 35) | 11.6 (638) | 10.8 (381) |
| [35, 50) | 22.5 (1,237) | 21.6 (761) |
| [50, 65) | 31.7 (1,743) | 33.9 (1,194) |
| [65, 80) | 31.1 (1,709) | 31.1 (1,094) |
| [80, 100) | 3.1 (171) | 2.6 (91) |
| Female | 63.7 (3,502) | 62.7 (2,763) |
| Race/ethnicity |  |  |
| NHW | 90.3 (4,802) | 87.5 (4,517) |
| NHB | 3.1 (164) | 3.1 (161) |
| Other | 6.6 (351) | 9.4 (486) |
| Marital status |  |  |
| Married | 55.9 (3,072) | 68.0 (3,242) |
| Unmarried | 28.5 (1,569) | 16.8 (802) |
| Unknown | 15.6 (857) | 15.2 (724) |
| Alcohol consumption (Ever/Never) | 79.9 (4,146) | 96.8 (4,328) |
| Smoking status (Ever/Never) | 37.7 (2,062) | 46.5 (2085) |
| Values represent proportion (sample size, n) unless otherwise specified. Percentages reported for non-missing individuals only. | | |
