# Supplementary material for: Epidemiologic Questionnaire (EPI-Q) – a scalable, app-based health survey linked to electronic health record and genotype data
Source: Epidemiol Health. 2023 Aug 8;45:e2023074. doi: 10.4178/epih.e2023074 (PMC10867525; doi:10.4178/epih.e2023074)
Supplement: Supplementary Material 7 — Factors associated with response to EPI-Q invitation [file epih-45-e2023074-Supplementary-7.docx]

**Supplementary Material 7. Factors associated with response to EPI-Q invitation**

We constructed two multivariable logistic regression models to describe factors associated with EPI-Q enrollment in greater detail (**Supplementary Material 8**). First, we fit a basic model (model 1) with age, sex, and race/ethnicity as covariates. We then considered an alternative model (model 2) which additionally included marital status and smoking. In both models, being female was associated with increased odds of EPI-Q participation while being Black was associated with lower odds of EPI-Q participation. In model 2, being married was associated with increased odds of EPI-Q participation while being a current smoker was associated with lower odds of EPI-Q participation.
