# Supplementary material for: Epidemiologic Questionnaire (EPI-Q) – a scalable, app-based health survey linked to electronic health record and genotype data
Source: Epidemiol Health. 2023 Aug 8;45:e2023074. doi: 10.4178/epih.e2023074 (PMC10867525; doi:10.4178/epih.e2023074)
Supplement: Supplementary Material 8 — Multivariable logistic regression models of response to survey vs nonresponse [file epih-45-e2023074-Supplementary-8.docx]

| **Supplementary Material 8**. Multivariable logistic regression models of response to survey vs nonresponse | | |
| --- | --- | --- |
|  | **OR estimate (95% CI)** | |
| **Variable** | **Model 1** | **Model 2** |
| Age | 1.00 (0.99, 1.00) | 1.00 (0.99, 1.00) |
| Female | **1.36** (1.27, 1.45) | **1.35** (1.27, 1.44) |
| Race/Ethnicity (ref = NHW) |  |  |
| NHB | **0.52** (0.43, 0.62) | **0.54** (0.45, 0.64) |
| Other | 0.97 (0.85, 1.10) | 0.95 (0.83, 1.09) |
| Married | - | **1.19** (1.11, 1.27) |
| Smoker (ref = Never) |  |  |
| Former | - | **0.89** (0.83, 0.96) |
| Current | - | **0.63** (0.57, 0.70) |
| *Abbreviations*: CI, confidence interval; NHB, Non-Hispanic Black; NHW, Non-Hispanic White; OR, odds ratio  Notes: Both models were limited to 44,265 individuals with nonmissing data for all variables included in model 2. Bolded point estimates represent statistical significance at the 95% confidence level. | | |
