# Supplementary material for: Epidemiologic Questionnaire (EPI-Q) – a scalable, app-based health survey linked to electronic health record and genotype data
Source: Epidemiol Health. 2023 Aug 8;45:e2023074. doi: 10.4178/epih.e2023074 (PMC10867525; doi:10.4178/epih.e2023074)
Supplement: Supplementary Material 9 — Proportion of cohort population by county in Michigan [file epih-45-e2023074-Supplementary-9.docx]

| **Supplementary Material 9**. Proportion of cohort population by county in Michigan | | | |
| --- | --- | --- | --- |
|  | **Cohort** | | |
|  | **EPI-Q** | **Combined Precision Health cohort** | **State*** |
| **County** | (n = 5,498) | (n = 90,076) | (n = 10,077,331) |
| Washtenaw | 32.1 | 22.9 | 3.7 |
| Wayne | 16.1 | 15.8 | 17.8 |
| Oakland | 9.2 | 8.7 | 12.6 |
| Livingston | 7.6 | 6.6 | 1.9 |
| Genesee | 3.4 | 4.7 | 4 |
| Ingham | 3.0 | 2.7 | 2.8 |
| Jackson | 2.3 | 3.6 | 1.6 |
| Lenawee | 2.1 | 2.7 | 1 |
| Monroe | 2.0 | 2.7 | 1.5 |
| Macomb | 1.5 | 2.1 | 8.7 |
| Kalamazoo | 1.4 | 1.5 | 2.6 |
| Saginaw | 1.3 | 1.8 | 1.9 |
| Kent | 1.3 | 1.3 | 6.5 |
| Calhoun | 1.1 | 1.4 | 1.3 |
| Bay | 0.9 | 1.1 | 1 |
| Ottawa | 0.8 | 0.7 | 2.9 |
| Clinton | 0.8 | 0.7 | 0.8 |
| Midland | 0.8 | 1.2 | 0.8 |
| Eaton | 0.7 | 1.0 | 1.1 |
| Grand Traverse | 0.7 | 0.7 | 0.9 |
| St. Clair | 0.6 | 0.9 | 1.6 |
| Emmet | 0.5 | 0.4 | 0.3 |
| Shiawassee | 0.5 | 0.9 | 0.7 |
| Berrien | 0.5 | 0.7 | 1.5 |
| Isabella | 0.5 | 0.5 | 0.6 |
| Lapeer | 0.5 | 0.6 | 0.9 |
| Muskegon | 0.5 | 0.7 | 1.7 |
| Hillsdale | 0.4 | 0.8 | 0.5 |
| Branch | 0.4 | 0.5 | 0.4 |
| St. Joseph | 0.4 | 0.4 | 0.6 |
| Leelanau | 0.3 | 0.2 | 0.2 |
| Van Buren | 0.3 | 0.5 | 0.8 |
| Tuscola | 0.3 | 0.5 | 0.5 |
| Alpena | 0.3 | 0.4 | 0.3 |
| Marquette | 0.3 | 0.3 | 0.7 |
| Cheboygan | 0.2 | 0.3 | 0.3 |
| Alcona | 0.2 | 0.2 | 0.1 |
| Clare | 0.2 | 0.4 | 0.3 |
| Gladwin | 0.2 | 0.3 | 0.3 |
| Wexford | 0.2 | 0.2 | 0.3 |
| Allegan | 0.2 | 0.4 | 1.2 |
| Charlevoix | 0.2 | 0.3 | 0.3 |
| Chippewa | 0.2 | 0.3 | 0.4 |
| Iosco | 0.2 | 0.3 | 0.3 |
| Sanilac | 0.2 | 0.3 | 0.4 |
| Ionia | 0.2 | 0.2 | 0.7 |
| Houghton | 0.2 | 0.1 | 0.4 |
| Huron | 0.2 | 0.3 | 0.3 |
| Ogemaw | 0.2 | 0.2 | 0.2 |
| Presque Isle | 0.2 | 0.2 | 0.1 |
| Barry | 0.1 | 0.2 | 0.6 |
| Gratiot | 0.1 | 0.4 | 0.4 |
| Manistee | 0.1 | 0.2 | 0.2 |
| Otsego | 0.1 | 0.3 | 0.2 |
| Benzie | 0.1 | 0.1 | 0.2 |
| Crawford | 0.1 | 0.1 | 0.1 |
| Mackinac | 0.1 | 0.1 | 0.1 |
| Antrim | 0.1 | 0.2 | 0.2 |
| Cass | 0.1 | 0.2 | 0.5 |
| Montmorency | 0.1 | 0.1 | 0.1 |
| Osceola | 0.1 | 0.1 | 0.2 |
| Arenac | 0.1 | 0.1 | 0.1 |
| Delta | 0.1 | 0.1 | 0.4 |
| Kalkaska | 0.1 | 0.1 | 0.2 |
| Lake | 0.1 | 0.1 | 0.1 |
| Mason | 0.1 | 0.1 | 0.3 |
| Mecosta | 0.1 | 0.1 | 0.4 |
| Montcalm | 0.1 | 0.2 | 0.7 |
| Roscommon | 0.1 | 0.3 | 0.2 |
| Missaukee | 0.0 | 0.1 | 0.1 |
| Newaygo | 0.0 | 0.1 | 0.5 |
| Ontonagon | 0.0 | 0.0 | 0.1 |
| Oscoda | 0.0 | 0.1 | 0.1 |
| Schoolcraft | 0.0 | 0.1 | 0.1 |
| Alger | 0.0 | 0.1 | 0.1 |
| Baraga | 0.0 | 0.0 | 0.1 |
| Dickinson | 0.0 | 0.0 | 0.3 |
| Luce | 0.0 | 0.0 | 0.1 |
| Oceana | 0.0 | 0.1 | 0.3 |
| Gogebic | 0.0 | 0.0 | 0.1 |
| Iron | 0.0 | 0.0 | 0.1 |
| Keweenaw | 0.0 | 0.0 | 0 |
| Menominee | 0.0 | 0.0 | 0.2 |
| * State of Michigan population estimates come from 2020 Census Demographics and Housing Survey | | | |
