# Supplementary material for: Epidemiologic Questionnaire (EPI-Q) – a scalable, app-based health survey linked to electronic health record and genotype data
Source: Epidemiol Health. 2023 Aug 8;45:e2023074. doi: 10.4178/epih.e2023074 (PMC10867525; doi:10.4178/epih.e2023074)
Supplement: Supplementary Material 10 — Comparison of UM Precision Health participants and EPI-Q participants based on data available in the electronic health record. [file epih-45-e2023074-Supplementary-10.docx]

| **Supplementary Material 10.** Comparison of UM Precision Health participants and EPI-Q participants based on data available in the electronic health record. | | |
| --- | --- | --- |
|  | **Precision Health** | **EPI-Q** |
| **N** | 90,076 | 5,801 |
| **Age (Years)** |  |  |
| *Range* | 15-106 | 18-96 |
| *Mean* | 57.6 | 55.9 |
| *Median* | 59.7 | 57.8 |
| **Sex** | % (n) |  |
| *Male* | 45.4 (40,910) | 36.2 (2,098) |
| *Female* | 54.6 (49,162) | 63.8 (3,703) |
| *Other* | 0.0 (4) | 0.0 (0) |
| *Unknown* | 0.0 (0) | 0.0 (0) |
| **Race** |  |  |
| *American Indian/Alaska Native* | 0.5 (494) | 0.5 (29) |
| *Asian* | 3.1 (2,777) | 2.5 (145) |
| *Black/African American* | 6.5 (5,855) | 3.2 (188) |
| *Native Hawaiian/Other Pacific Islander* | 0.1 (89) | 0.1 (4) |
| *White* | 85.6 (77,117) | 90.8 (5,268) |
| *Other* | 2.3 (2,105) | 1.8 (102) |
| *Patient Refused* | 0.4 (390) | 0.3 (16) |
| *Unknown* | 1.4 (1,249) | 0.8 (49) |
| **Ethnicity** |  |  |
| *Hispanic/Latino* | 3.0 (2,698) | 2.5 (147) |
| *Non-Hispanic/Latino* | 93.6 (84,311) | 94.5 (5,482) |
| *Patient Refused* | 0.5 (445) | 0.4 (26) |
| *Unknown* | 2.9 (2,622) | 2.5 (146) |
| **Race/Ethnicity** |  |  |
| *White, Non-Hispanic* | 84.1 (75,788) | 89.4 (5,185) |
| *Black, Non-Hispanic* | 6.4 (5,768) | 3.2 (184) |
| *Other, Non-Hispanic* | 6.5 (5,823) | 4.9 (285) |
| *Hispanic* | 3.0 (2,698) | 2.5 (147) |
| **BMI** |  |  |
| *<18.5* | 1.6 (1,428) | 1.1 (66) |
| *18.5-24.9* | 25.0 (22,517) | 24.5 (1,422) |
| *25.0-29.9* | 30.6 (27,564) | 30.5 (1,771) |
| *30.0-34.9* | 21.5 (19,391) | 22.0 (1,276) |
| *35.0-39.9* | 11.2 (10,103) | 11.2 (648) |
| *>40* | 8.9 (8,015) | 9.9 (572) |
| *Unknown* | 1.2 (1,058) | 0.8 (46) |
| **Marital Status** |  |  |
| *Married* | 51.0 (45,940) | 55.4 (3,213) |
| *Unmarried* | 33.6 (30,247) | 29.0 (1,680) |
| *Unknown* | 15.4 (13,889) | 15.7 (908) |
| **Drinking Status** |  |  |
| *Yes* | 52.5 (47,289) | 59.7 (3,466) |
| *No* | 31.5 (28,378) | 22.0 (1,277) |
| *Not Asked* | 2.1 (1,868) | 0.8 (46) |
| *Unknown* | 13.9 (12,550) | 17.4 (1,012) |
| **Smoking Status** |  |  |
| *Current* | 9.5 (8,530) | 4.5 (263) |
| *Former* | 34.4 (30,965) | 31.8 (1,843) |
| *Never* | 54.9 (49,415) | 63.1 (3,658) |
| *Unknown* | 1.3 (1,174) | 0.6 (37) |
