# Supplementary material for: Epidemiologic Questionnaire (EPI-Q) – a scalable, app-based health survey linked to electronic health record and genotype data
Source: Epidemiol Health. 2023 Aug 8;45:e2023074. doi: 10.4178/epih.e2023074 (PMC10867525; doi:10.4178/epih.e2023074)
Supplement: Supplementary Material 11 — Schematic describing the recruitment process from the Michigan Genomics Initiative into the Epidemiological Questionnaire. [file epih-45-e2023074-Supplementary-11.docx]

**Supplementary Material 11**. Schematic describing the recruitment process from the Michigan Genomics Initiative into the Epidemiological Questionnaire.
