# Supplementary material for: Epidemiologic Questionnaire (EPI-Q) – a scalable, app-based health survey linked to electronic health record and genotype data
Source: Epidemiol Health. 2023 Aug 8;45:e2023074. doi: 10.4178/epih.e2023074 (PMC10867525; doi:10.4178/epih.e2023074)
Supplement: Supplementary Material 12 — The number of questions in EPI-Q by baseline and optional module and the proportion of questions that also appear in the UK Biobank questionnaire. The gray bars represent the number of questions in the EPI-Q module while the orange portion of each bar represents the proportion of questions that overlap with the UK Biobank. [file epih-45-e2023074-Supplementary-12.docx]

**Supplementary Material 12**. The number of questions in EPI-Q by baseline and optional module and the proportion of questions that also appear in the UK Biobank questionnaire. The gray bars represent the number of questions in the EPI-Q module while the orange portion of each bar represents the proportion of questions that overlap with the UK Biobank.
