# Supplementary material for: Epidemiologic Questionnaire (EPI-Q) – a scalable, app-based health survey linked to electronic health record and genotype data
Source: Epidemiol Health. 2023 Aug 8;45:e2023074. doi: 10.4178/epih.e2023074 (PMC10867525; doi:10.4178/epih.e2023074)
Supplement: Supplementary Material 13 — Modules, their domains, and number of questions in baseline and optional EPI-Q surveys [file epih-45-e2023074-Supplementary-13.docx]

| **Supplementary Material 13**. Modules, their domains, and number of questions in baseline and optional EPI-Q surveys | | |
| --- | --- | --- |
| **BASELINE MODULES** | | |
| **Module** | **Domain** | **Questions (N)** |
| Home and Personal Details | Housing | 5 |
|  | Employment | 5 |
|  | Work context | 3 |
|  | Transportation | 2 |
|  | Education | 2 |
|  | Marital status | 1 |
|  | Income | 1 |
|  | Age | 1 |
| Physical Activity | Physical activity | 9 |
| Social and recreational activity | Leisure time | 10 |
|  | Sleep | 7 |
|  | Mobile phone use | 4 |
|  | Transportation | 2 |
| Smoking | Smoking | 25 |
| Alcohol | Alcohol | 11 |
| Personal and family traits | Parent | 14 |
|  | Family origin | 4 |
|  | Trait | 4 |
|  | Siblings | 4 |
|  | Sun exposure | 3 |
|  | Race/ethnicity | 2 |
|  | Infancy | 2 |
|  | Family | 1 |
| Feelings and mood | Mental health | 17 |
|  | Depression | 9 |
|  | Life satisfaction | 6 |
| Sexual orientation and history | Sexual history | 3 |
|  | Sexual orientation | 2 |
| Personal health | Women's health | 27 |
|  | Cardiovascular health | 7 |
|  | Men's health | 4 |
|  | Allergies | 4 |
|  | Diabetes | 4 |
|  | Bone health | 3 |
|  | Gender | 2 |
|  | Chronic condition | 2 |
|  | Vision | 2 |
|  | Lung health | 2 |
|  | Medications | 2 |
|  | Sex | 1 |
|  | Overall health | 1 |
|  | Health insurance | 1 |
|  | Oral health | 1 |
|  | Balance | 1 |
|  | Weight | 1 |
|  | Pains | 1 |
|  | Surgery | 1 |
| Cancer screening and history | Family cancer history | 51 |
|  | Personal cancer history | 42 |
|  | Cancer screening | 18 |
| Hearing | Hearing | 8 |
| **BASELINE TOTAL** |  | **351** |
|  |  |  |
| **OPTIONAL MODULES** | | |
| **Module** | **Domain** | **Questions (N)** |
| Alcohol | Alcohol | 6 |
| COVID-19 | COVID-19 | 30 |
| Depression | Depression | 12 |
| Diet | Diet | 32 |
| Healthcare access and utilization | Financial toxicity | 12 |
|  | Healthcare utilization | 12 |
|  | Barriers to healthcare | 9 |
|  | Care avoided due to cost | 8 |
|  | Unmet social needs | 8 |
|  | Medicine avoided due to cost | 5 |
|  | Insurance churning | 5 |
|  | Healthcare finances | 1 |
|  | Health literacy | 1 |
|  | Recent treatment | 1 |
| Life meaning | Global coherence | 3 |
|  | Individual coherence | 3 |
|  | Subjective significance | 3 |
|  | Objective significance | 3 |
|  | Mission | 3 |
|  | Purposes | 3 |
|  | Goals | 3 |
| Life satisfaction, stress, and anxiety | Anxiety | 7 |
|  | Life satisfaction | 5 |
|  | Perceived stress | 4 |
| Occupational exposure | Work context | 42 |
|  | Occupational exposure | 15 |
| Pain | Pain | 24 |
| Physical activity | Physical activity | 24 |
| Substance use | Smoking | 11 |
|  | Drugs | 11 |
| Vision | Vision | 34 |
| **OPTIONAL TOTAL** |  | **340** |
