# Supplementary material for: Epidemiologic Questionnaire (EPI-Q) – a scalable, app-based health survey linked to electronic health record and genotype data
Source: Epidemiol Health. 2023 Aug 8;45:e2023074. doi: 10.4178/epih.e2023074 (PMC10867525; doi:10.4178/epih.e2023074)
Supplement: Supplementary Material 14 — Highlighting EPI-Q survey instruments that are less common or represent upcoming areas of research [file epih-45-e2023074-Supplementary-14.docx]

**Supplementary Material 14. Highlighting EPI-Q survey instruments that are less common or represent upcoming areas of research**

Occupational exposures result in millions of deaths and hundreds of millions of non-fatal injuries globally each year (14). To better understand how one’s workplace may impact their health, we incorporated the Work Context questionnaire from the Occupational Information Network (O*NET), a program sponsored by the US Department of Labor (15), as an optional occupational exposure module. Not only does the time spent at work represent a crucial window of exposure but also represents an opportunity for intervention.

Financial toxicity represents financial costs including out-of-pocket costs, loss of income, and caregiver burden associated with healthcare utilization (e.g., cancer treatment) (16). We implement a modified version of the Comprehensive Score for financial toxicity (COST) instrument, developed and validated (16) to assess financial distress in cancer patients, though recently it has become more broadly used (e.g., Crohn’s disease (17)).

Dr. Tyler VanderWeele, program director of the Human Flourishing Program at Harvard’s Institute for Quantitative Social Science, has argued that the health sciences tend to focus on outcomes that are too narrow in their scope, especially in the context of measures of positive affect (18). Though considerable research examines psychosocial constructs such as life purpose, life satisfaction, and happiness, VanderWeele suggests that we instead adopt a broader measure – human well-being and flourishing. To that end, the Human Flourishing Program consolidated 21-items across three domains into a single instrument, called the Comprehensive Measure of Meaning (CMM) (19), which we now deploy as our life meaning module.
