# Supplementary material for: Epidemiologic Questionnaire (EPI-Q) – a scalable, app-based health survey linked to electronic health record and genotype data
Source: Epidemiol Health. 2023 Aug 8;45:e2023074. doi: 10.4178/epih.e2023074 (PMC10867525; doi:10.4178/epih.e2023074)
Supplement: Supplementary Material 15 — Proportion of participants who completed EPI-Q by baseline and optional modules. Baseline modules were required for participants to receive the incentive; there was no incentive for completing optional modules. [file epih-45-e2023074-Supplementary-15.docx]

**Supplementary Material 15**. Proportion of participants who completed EPI-Q by baseline and optional modules. Baseline modules were required for participants to receive the incentive; there was no incentive for completing optional modules.
