# Supplementary material for: Epidemiologic Questionnaire (EPI-Q) – a scalable, app-based health survey linked to electronic health record and genotype data
Source: Epidemiol Health. 2023 Aug 8;45:e2023074. doi: 10.4178/epih.e2023074 (PMC10867525; doi:10.4178/epih.e2023074)
Supplement: Supplementary Material 16 — Average time-to-complete (95% confidence interval; in minutes) baseline and optional modules in the Epidemiological Questionnaire (EPI-Q). Based on pilot data from 601 respondents from 5,000 initial invitations after removing individual responses with completion times that qualify as outliers (IQR±1.5×IQR). [file epih-45-e2023074-Supplementary-16.docx]

**Supplementary Material 16**. Average time-to-complete (95% confidence interval; in minutes) baseline and optional modules in the Epidemiological Questionnaire (EPI-Q). Based on pilot data from 601 respondents from 5,000 initial invitations after removing individual responses with completion times that qualify as outliers ($\mathrm{IQR}\pm1.5\times\mathrm{IQR}$).
