# Supplementary material for: Epidemiologic Questionnaire (EPI-Q) – a scalable, app-based health survey linked to electronic health record and genotype data
Source: Epidemiol Health. 2023 Aug 8;45:e2023074. doi: 10.4178/epih.e2023074 (PMC10867525; doi:10.4178/epih.e2023074)
Supplement: Supplementary Material 17 — Comparison of self-reported and EHR-recorded concordance [file epih-45-e2023074-Supplementary-17.docx]

| **Supplementary Material 17**. Comparison of self-reported and EHR-recorded concordance | | | |
| --- | --- | --- | --- |
|  | **EHR-recorded sex** | |  |
| **Self-reported sex** | Female | Male | Total |
| Female | 2,746 | 17 | 2,763 |
| Male | 12 | 1,630 | 1,642 |
| Total | 2,758 | 1,647 | 4,405 |
| Self-reported sex asked about biological sex assigned at birth.  Cohen's kappa among non-missing = 0.986 | | | |
