# Supplementary material for: Epidemiologic Questionnaire (EPI-Q) – a scalable, app-based health survey linked to electronic health record and genotype data
Source: Epidemiol Health. 2023 Aug 8;45:e2023074. doi: 10.4178/epih.e2023074 (PMC10867525; doi:10.4178/epih.e2023074)
Supplement: Supplementary Material 18 — Comments regarding discrepancy between EHR-defined and self-reported history of cancer [file epih-45-e2023074-Supplementary-18.docx]

**Supplementary Material 18. Comments regarding discrepancy between EHR-defined and self-reported history of cancer**

| **Supplementary Material 19**. Concordance between self-reported history of cancer and EHR-recorded diagnosis of cancer | | | |
| --- | --- | --- | --- |
|  | **EHR-recorded cancer** | |  |
| **Self-reported history of cancer** | Had cancer | No cancer | Total |
| Had cancer | 1,789 | 61 | 1,850 |
| No cancer | 1,191 | 2,090 | 3,281 |
| Missing | 202 | 165 | 367 |
| Total | 2,182 | 2,316 | 5,498 |
| Qualifying cancer codes from the EHR are presented in **Table S9**. Cohen's kappa among non-missing = 0.668 | | | |

A non-negligible number of participants (1,191 among 3,281; 36.3%) self-reported that they did not have cancer while they were defined has having a history of cancer based on their EHR. This section presents related comments in addition to the proposed explanations for the discrepancy discussed in the main text.

Misclassification can exist both from incomplete EHR (not designed for research) across the US’ fragmented healthcare system (20) and also from recall and self-reporting bias from patient-provided information (e.g., as in a survey like EPI-Q); both of which are well documented (21,22).

UM Precision Health cohorts only have access to a participant’s Michigan Medicine EHR. As a result, it is expected for there to be individuals who self-report a history of cancer that may not appear on their Michigan Medicine EHR. In the future, EPI-Q has mechanisms that will allow participants to share third-party EHR data (e.g., from their primary care healthcare provider), which would improve the completeness of the EHR data.

EHR-defined traits vary in their level of misclassification. For a trait like cancer, it is expected that that is a relatively low change for there to be a false positive. However, this misclassification could be much higher for other conditions. Because of how EHR data are collected, it is not practicably possible to fix in the design stage, but there are ways to account for this and describe robustness of results to misclassification in the analysis phase. For example, methods like double or multi-wave sampling strategies that could be employed in an attempt to improve accuracy of association estimation due to, at least in part, the presence of misclassification (23–25).

Investigation into EHR-defined vs. self-reported cancer status in EPI-Q

After restricting to the initial occurrence of a given phecode, we found that among the 3,193 individuals who were defined as having cancer using their whole EHR, 99.7% (n = 3,182) retained their cancer history status after restricting EHR to age 18+. Of those 11 individuals who had their only initial cancer diagnosis during childhood, there were 10 different cancer diagnoses [phecode]: chemotherapy [197] (x2), cancer of esophagus [150], neoplasm of unspecified nature of digestive system [158], other non-epithelial cancer of skin [172.2], malignant neoplasm of kidney, except pelvis [189.11], cancer of bladder [189.2], malignant and unknown neoplasms of brain and nervous system [191], malignant neoplasm, other [195.1], myeloproliferative disease [200], myeloid leukemia, acute [204.21]. This indicates that the discrepancy seen is not attributable to high rates of childhood cancer diagnoses that participants either do not remember or do not consider cancer diagnoses.

We report the proportion of individuals self-reporting history of cancer via the EPI-Q survey that have a recorded EHR diagnosis by ICD-derived phecode in **Supplementary Material 20**.
