# Supplementary material for: Epidemiologic Questionnaire (EPI-Q) – a scalable, app-based health survey linked to electronic health record and genotype data
Source: Epidemiol Health. 2023 Aug 8;45:e2023074. doi: 10.4178/epih.e2023074 (PMC10867525; doi:10.4178/epih.e2023074)
Supplement: Supplementary Material 20 — Occurrence of qualifying cancer International Classification of Disease (ICD)-derived traits (phecodes as first described by Denny and colleagues (26,27)) and the proportion of individuals who self-reported history of cancer [file epih-45-e2023074-Supplementary-20.docx]

| **Supplementary Material 20**. Occurrence of qualifying cancer International Classification of Disease (ICD)-derived traits (phecodes as first described by Denny and colleagues (26,27)) and the proportion of individuals who self-reported history of cancer | | | |
| --- | --- | --- | --- |
| **Cancer trait [phecode]** | **EHR-recorded cases** | **Self-reported cancer** | **% with corresponding phecode who self-reported cancer** |
| Cancer of the gums [145.4] | 9 | 9 | 100 |
| Cancer of nasopharynx [149.2] | 9 | 9 | 100 |
| Cancer of hypopharynx [149.3] | 3 | 3 | 100 |
| Malignant neoplasm of unspecified male genital organ [187.1] | 6 | 6 | 100 |
| Hodgkin's disease [201] | 21 | 21 | 100 |
| Myeloid leukemia, chronic [204.22] | 7 | 7 | 100 |
| Malignant neoplasm of small intestine, including duodenum [159.2] | 27 | 26 | 96.3 |
| Cancer of tongue [145.2] | 53 | 51 | 96.2 |
| Malignant neoplasm of retroperitoneum and peritoneum [159.4] | 25 | 24 | 96 |
| Hx of malignant neoplasm of oral cavity and pharynx [149.5] | 46 | 44 | 95.7 |
| Secondary malignancy of bone [198.6] | 75 | 71 | 94.7 |
| Secondary malignant neoplasm of liver [198.4] | 55 | 52 | 94.5 |
| Cancer of oropharynx [149.1] | 33 | 31 | 93.9 |
| Secondary malignancy of respiratory organs [198.2] | 61 | 57 | 93.4 |
| Secondary malignancy of lymph nodes [198.1] | 339 | 315 | 92.9 |
| Cancer of larynx [149.4] | 13 | 12 | 92.3 |
| Cancer of intrathoracic organs [164] | 13 | 12 | 92.3 |
| Malignant neoplasm of other and ill-defined sites within the digestive organs and peritoneum [159] | 90 | 83 | 92.2 |
| Cancer of larynx, pharynx, nasal cavities [149] | 91 | 83 | 91.2 |
| Secondary malignant neoplasm of digestive systems [198.3] | 57 | 52 | 91.2 |
| Cancer of mouth [145] | 99 | 90 | 90.9 |
| Secondary malignant neoplasm [198] | 491 | 445 | 90.6 |
| Cancer of prostate [185] | 332 | 300 | 90.4 |
| Malignant neoplasm of head, face, and neck [195.3] | 69 | 62 | 89.9 |
| Bone cancer [170.1] | 59 | 53 | 89.8 |
| Malignant neoplasm of uterus [182] | 99 | 88 | 88.9 |
| Malignant neoplasm of bladder [189.21] | 126 | 112 | 88.9 |
| Lymphosarcoma [202.23] | 9 | 8 | 88.9 |
| Large cell lymphoma [202.24] | 9 | 8 | 88.9 |
| Malignant neoplasm of other urinary organs [189.4] | 42 | 37 | 88.1 |
| Malignant neoplasm of gallbladder and extrahepatic bile ducts [159.3] | 8 | 7 | 87.5 |
| Cancer of other male genital organs [187] | 31 | 27 | 87.1 |
| Malignant neoplasm, other [195.1] | 1,492 | 1,290 | 86.5 |
| Reticulosarcoma [202.22] | 22 | 19 | 86.4 |
| Cancer, suspected or other [195] | 1,533 | 1,314 | 85.7 |
| Secondary malignancy of brain/spine [198.5] | 34 | 29 | 85.3 |
| Cancer of bladder [189.2] | 142 | 121 | 85.2 |
| Malignant neoplasm of female breast [174.11] | 485 | 412 | 84.9 |
| Cancer of bronchus; lung [165.1] | 65 | 55 | 84.6 |
| Breast cancer [female] [174.1] | 493 | 416 | 84.4 |
| Cancer within the respiratory system [165] | 70 | 59 | 84.3 |
| Malignant neoplasm of testis [187.2] | 19 | 16 | 84.2 |
| Breast cancer [174] | 505 | 424 | 84 |
| Thyroid cancer [193] | 123 | 102 | 82.9 |
| Melanomas of skin [172.11] | 279 | 231 | 82.8 |
| Cancer of urinary organs (incl. kidney and bladder) [189] | 238 | 197 | 82.8 |
| Cancer of of nasal cavities [149.9] | 17 | 14 | 82.4 |
| Malignant neoplasm of ovary and other uterine adnexa [184.1] | 74 | 61 | 82.4 |
| Melanomas of skin, dx or hx [172.1] | 293 | 241 | 82.3 |
| Cancer of bone and connective tissue [170] | 168 | 138 | 82.1 |
| Cervical cancer [180.1] | 61 | 50 | 82 |
| Malignant neoplasm of kidney, except pelvis [189.11] | 100 | 82 | 82 |
| Non-Hodgkins lymphoma [202.2] | 149 | 122 | 81.9 |
| Malignant neoplasm of liver, primary [155.1] | 27 | 22 | 81.5 |
| Cancer of major salivary glands [145.3] | 32 | 26 | 81.2 |
| Lymphoid leukemia, chronic [204.12] | 21 | 17 | 81 |
| Colon cancer [153.2] | 136 | 110 | 80.9 |
| Cancer of stomach [151] | 26 | 21 | 80.8 |
| Cancer of kidney and renal pelvis [189.1] | 109 | 88 | 80.7 |
| Neuroendocrine tumors [209] | 31 | 25 | 80.6 |
| Cancer of liver and intrahepatic bile duct [155] | 41 | 33 | 80.5 |
| Lymphoid leukemia [204.1] | 30 | 24 | 80 |
| Cancer of connective tissue [170.2] | 129 | 103 | 79.8 |
| Cancer of other lymphoid, histiocytic tissue [202] | 170 | 135 | 79.4 |
| Malignant neoplasm of rectum, rectosigmoid junction, and anus [153.3] | 101 | 80 | 79.2 |
| Nodular lymphoma [202.21] | 77 | 61 | 79.2 |
| Acquired absence of breast [175] | 271 | 214 | 79 |
| Malignant neoplasm of ovary [184.11] | 57 | 45 | 78.9 |
| Cancer of esophagus [150] | 23 | 18 | 78.3 |
| Pancreatic cancer [157] | 35 | 27 | 77.1 |
| Squamous cell carcinoma [172.22] | 257 | 198 | 77 |
| Secondary malignant neoplasm of skin [198.7] | 34 | 26 | 76.5 |
| Other non-epithelial cancer of skin [172.2] | 766 | 580 | 75.7 |
| Skin cancer [172] | 894 | 674 | 75.4 |
| Malignant neoplasm of renal pelvis [189.12] | 12 | 9 | 75 |
| Colorectal cancer [153] | 182 | 135 | 74.2 |
| Cancer of other female genital organs [184] | 123 | 90 | 73.2 |
| Cancer of brain [191.11] | 51 | 37 | 72.5 |
| Cancer of brain and nervous system [191.1] | 61 | 44 | 72.1 |
| Basal cell carcinoma [172.21] | 436 | 314 | 72 |
| Lymphoid leukemia, acute [204.11] | 14 | 10 | 71.4 |
| Carcinoma in situ of skin [172.3] | 134 | 93 | 69.4 |
| Cancer of eye [190] | 16 | 11 | 68.8 |
| Myeloid leukemia [204.2] | 22 | 15 | 68.2 |
| Monocytic leukemia [204.3] | 3 | 2 | 66.7 |
| Cancer of other female genital organs (excluding uterus and ovary) [184.2] | 53 | 35 | 66 |
| Leukemia [204] | 96 | 62 | 64.6 |
| Cancer of other endocrine glands [194] | 32 | 20 | 62.5 |
| Multiple myeloma [204.4] | 42 | 26 | 61.9 |
| Myeloid leukemia, acute [204.21] | 17 | 10 | 58.8 |
| Malignant and unknown neoplasms of brain and nervous system [191] | 96 | 55 | 57.3 |
| Neoplasm of unspecified nature of digestive system [158] | 189 | 108 | 57.1 |
| Chemotherapy [197] | 2,057 | 1,066 | 51.8 |
| Polycythemia vera [200.1] | 33 | 16 | 48.5 |
| Myeloproliferative disease [200] | 158 | 76 | 48.1 |
| Radiotherapy [196] | 1,758 | 813 | 46.2 |
| Cervical cancer and dysplasia [180] | 246 | 87 | 35.4 |
| Cervical intraepithelial neoplasia [CIN] [Cervical dysplasia] [180.3] | 207 | 51 | 24.6 |
| Neurofibromatosis [199.4] | 6 | 1 | 16.7 |
