# Supplementary material for: Epidemiologic Questionnaire (EPI-Q) – a scalable, app-based health survey linked to electronic health record and genotype data
Source: Epidemiol Health. 2023 Aug 8;45:e2023074. doi: 10.4178/epih.e2023074 (PMC10867525; doi:10.4178/epih.e2023074)
Supplement: Supplementary Material 21 — Time since most recent initial cancer diagnosis by individual. [file epih-45-e2023074-Supplementary-21-23.docx]

It is possible that there are certain cancer traits that are included in our list of qualifying cancer phecodes that participants do not recognize as cancer. Two potential traits include polycythemia vera [pheode 200.1] and cervical intraepithelial neoplasia [phecode 180.3]. Of the 3,182 individuals with an initial cancer diagnosis in adulthood, 223 had an initial adult diagnosis of either polycythemia vera or cervical intraepithelial neoplasia. All of these 223 individuals had other qualifying initial cancer diagnoses during adulthood. This suggests that even if these traits were excluded as qualifying cancer traits, these individuals would still have been classified as having cancer by their EHR. While this avoidable in this instance, it may not be in other instances.

It is possible that there are individuals have only a single qualifying cancer phecode, and because of the single diagnosis, may not remember or recognize a cancer diagnosis. However, of the 3,182 individual with an initial cancer diagnosis in adulthood, 5.5% (n = 180) had only a single qualifying cancer phecode on their EHR.

It is possible that a significant amount of time has passed since an individual’s most recent initial cancer diagnosis, which could lead to a decreased likelihood of self-reporting cancer via EPI-Q. In **Supplementary Material 21**, we plot the time since most recent initial cancer diagnosis for all individuals with an EHR-defined cancer diagnosis.

**Supplementary Material 21**. Time since most recent initial cancer diagnosis by individual.

In **Supplementary Material 22**, we plotted the proportion of EHR-defined cancer patients self-reporting cancer by decile of time since most recent initial cancer diagnosis. We found that as time since most recent cancer diagnosis increases, the proportion of people self-reporting history decreases from 68% to 41%.

**Supplementary Material 22**. Proportion of EHR-defined cancer patients who self-reported history of cancer via EPI-Q by decile of time since most recent initial cancer diagnosis.

We also ran a series of logistic regression models of the form

$$logit\left( \mathrm{Cance}r_{self-report}=1 | \mathrm{time}, \boldsymbol{X} \right)=\beta_{0}+\beta_{\mathrm{time}}\mathrm{time}+\boldsymbol{\beta}_{\boldsymbol{X}}\boldsymbol{X}$$

where $\mathrm{Cance}r_{self-report}$ is an indicator variable corresponding to whether the individual self-reported a history of cancer (1) or not (0) in the survey, $\mathrm{time}$ is a continuous variable corresponding to time (in years) from most recent initial cancer diagnosis in the EHR to age at survey completion (**Supplementary Material 21**) and $\boldsymbol{X}$ are covariates. We compared a model without covariates to models with covariate sets sex and sex and number of unique qualifying cancer phecodes. The log-odds (i.e., beta coefficient) and p-value for the covariates are summarized in **Supplementary Material 23**. The results show that the time since most recent cancer diagnosis is negatively associated with self-reported history of cancer. After accounting for the number of unique cancer phecodes on an individual’s EHR, this association remains weakly negative and is not statistically significant. Being male and having more unique cancer phecodes is significantly positively associated with self-reported history of cancer. This suggests that there are female-specific cancers which an individual may be less likely to consider or self-report has history of cancer. Modeling quantity and density of EHR data in the form of number of encounters, number of codes, and time since relevant diagnoses are important and can serve as indicators of potential misclassification and reporting bias.

| **Supplementary Material 23**. Logistic regression models for self-reported history of cancer under three sets of covariates. | | | |
| --- | --- | --- | --- |
|  | Unadjusted | Sex-adjusted | Sex- and number of unique cancer phecodes-adjusted |
| Time since most recent initial cancer diagnosis (years) | **-0.095**  (5.96E-16) | **-0.087**  (1.48E-13) | -0.010  (4.78E-01) |
| Sex (Male = 1) | - | **0.570**  (4.38E-14) | **0.617**  (7.22E-12) |
| Number of unique cancer phecodes | - | - | **0.466**  (3.33E-143) |
| Point estimates are bolded if they are statistically significantly different from 0 at the 95% significance level. | | | |

Ultimately, while the inclusion of some of codes like those for polycythemia vera and cervical intraepithelial neoplasia could inflate the EHR-defined history of cancer count, their inclusion could reduce the agreement between EHR-defined and self-reported cancer status. In this case however, because virtually all individuals have multiple qualifying cancer diagnoses, the impact of the inclusion of these codes is minimal. Future work looking at self-reporting by EHR-defined cancer trait would need to be aware of these nuances and think carefully through which codes to include in defining a cancer diagnosis. EHR-based cancer research needs to better understand how misclassification varies by cancer type and be careful when assuming an individual’s cancer history is accurately reflected in their health record.
