# Supplementary material for: Epidemiologic Questionnaire (EPI-Q) – a scalable, app-based health survey linked to electronic health record and genotype data
Source: Epidemiol Health. 2023 Aug 8;45:e2023074. doi: 10.4178/epih.e2023074 (PMC10867525; doi:10.4178/epih.e2023074)
Supplement: Supplementary Material 25 — Responses to financial toxicity questions by self-reported history of cancer. [file epih-45-e2023074-Supplementary-25.docx]

**Supplementary Material 25**. Responses to financial toxicity questions by self-reported history of cancer.
