# Supplementary material for: Epidemiologic Questionnaire (EPI-Q) – a scalable, app-based health survey linked to electronic health record and genotype data
Source: Epidemiol Health. 2023 Aug 8;45:e2023074. doi: 10.4178/epih.e2023074 (PMC10867525; doi:10.4178/epih.e2023074)
Supplement: Supplementary Material 26 — Comparison of average life meaning scores by domain and overall by self-reported history of cancer [file epih-45-e2023074-Supplementary-26.docx]

| **Supplementary Material 26.** Comparison of average life meaning scores by domain and overall by self-reported history of cancer | | | | |
| --- | --- | --- | --- | --- |
|  |  | Self-reported cancer history | |  |
| Life meaning trait | n | No n = 3,281 | Yes n = 1,850 | P-value^†^ |
| Overall life meaning | 4,122 | 5..09 (1.32) | 5.35 (1.14) | 4.98E-11 |
| Cognitive coherence | 4,038 | 5.24 (1.43) | 5.51 (1.23) | 8.20E-10 |
| Affective significance | 4,091 | 5.14 (1.43) | 5.47 (1.15) | 1.36E-15 |
| Motivational purposive directive | 4,049 | 4.94 (1.45) | 5.16 (1.30) | 4.70E-07 |
| Each trait is measured as the average of the answers to multiple questions, which were translated to a numeric score from 1 to 7. ^†^P-value corresponds to Welch’s two-sample t-test. | | | | |
