# Supplementary material for: Epidemiologic Questionnaire (EPI-Q) – a scalable, app-based health survey linked to electronic health record and genotype data
Source: Epidemiol Health. 2023 Aug 8;45:e2023074. doi: 10.4178/epih.e2023074 (PMC10867525; doi:10.4178/epih.e2023074)
Supplement: Supplementary Material 28 — Manhattan plot summarizing p-values from life meaning GWAS of 2,433 EPI-Q participants who completed at least one question in the corresponding module. [file epih-45-e2023074-Supplementary-28.docx]

**Supplementary Material 28**. Manhattan plot summarizing p-values from life meaning GWAS of 2,433 EPI-Q participants who completed at least one question in the corresponding module.
