# Supplementary material for: Epidemiologic Questionnaire (EPI-Q) – a scalable, app-based health survey linked to electronic health record and genotype data
Source: Epidemiol Health. 2023 Aug 8;45:e2023074. doi: 10.4178/epih.e2023074 (PMC10867525; doi:10.4178/epih.e2023074)
Supplement: Supplementary References [file epih-45-e2023074-Supplementary-References.docx]

**References**

1. Kim E, Rubinstein SM, Nead KT, Wojcieszynski AP, Gabriel PE, Warner JL. The Evolving Use of Electronic Health Records (EHR) for Research. Semin Radiat Oncol. 2019 Oct;29(4):354–61.

2. Chan KS, Fowles JB, Weiner JP. Review: Electronic Health Records and the Reliability and Validity of Quality Measures: A Review of the Literature. Med Care Res Rev. 2010 Oct;67(5):503–27.

3. Palestine AG, Merrill PT, Saleem SM, Jabs DA, Thorne JE. Assessing the Precision of ICD-10 Codes for Uveitis in 2 Electronic Health Record Systems. JAMA Ophthalmol. 2018 Oct 1;136(10):1186.

4. Castro VM, Minnier J, Murphy SN, Kohane I, Churchill SE, Gainer V, et al. Validation of Electronic Health Record Phenotyping of Bipolar Disorder Cases and Controls. Am J Psychiatry. 2015 Apr;172(4):363–72.

5. Beesley LJ, Mukherjee B. Statistical inference for association studies using electronic health records: handling both selection bias and outcome misclassification. Biometrics. 2020 Dec 3;biom.13400.

6. Weiskopf NG, Weng C. Methods and dimensions of electronic health record data quality assessment: enabling reuse for clinical research. J Am Med Inform Assoc. 2013 Jan 1;20(1):144–51.

7. Meystre S, Haug PJ. Natural language processing to extract medical problems from electronic clinical documents: Performance evaluation. J Biomed Inform. 2006 Dec;39(6):589–99.

8. Marafino BJ, Park M, Davies JM, Thombley R, Luft HS, Sing DC, et al. Validation of Prediction Models for Critical Care Outcomes Using Natural Language Processing of Electronic Health Record Data. JAMA Netw Open. 2018 Dec 21;1(8):e185097.

9. Botsis T, Hartvigsen G, Chen F, Weng C. Secondary Use of EHR: Data Quality Issues and Informatics Opportunities. Summit Transl Bioinforma. 2010 Mar 1;2010:1–5.

10. Beesley LJ, Salvatore M, Fritsche LG, Pandit A, Rao A, Brummett C, et al. The emerging landscape of health research based on biobanks linked to electronic health records: Existing resources, statistical challenges, and potential opportunities. Stat Med. 2020 Mar 15;39(6):773–800.

11. Million Veteran Program (MVP) [Internet]. [cited 2021 Apr 12]. Available from: https://www.research.va.gov/mvp/

12. National Institutes of Health (NIH) [Internet]. National Institutes of Health (NIH) — All of Us. 2020 [cited 2021 Apr 12]. Available from: https://allofus.nih.gov/future-health-begins-all-us

13. Sudlow C, Gallacher J, Allen N, Beral V, Burton P, Danesh J, et al. UK Biobank: An Open Access Resource for Identifying the Causes of a Wide Range of Complex Diseases of Middle and Old Age. PLOS Med. 2015 Mar 31;12(3):e1001779.

14. University of Michigan Center for Occupational Health and Safety Engineering. Why Study Occupational Health? [Internet]. Available from: https://cohse.umich.edu/about-us/why-study-occupational-health/

15. About O*NET at O*NET Resource Center [Internet]. [cited 2022 Jul 2]. Available from: https://www.onetcenter.org/overview.html

16. de Souza JA, Yap BJ, Wroblewski K, Blinder V, Araújo FS, Hlubocky FJ, et al. Measuring financial toxicity as a clinically relevant patient-reported outcome: The validation of the COmprehensive Score for financial Toxicity (COST): Measuring Financial Toxicity. Cancer. 2017 Feb 1;123(3):476–84.

17. Voit A, Cross RK, Bellavance E, Bafford AC. Financial Toxicity in Crohn’s Disease. J Clin Gastroenterol. 2019 Nov;53(10):e438–43.

18. VanderWeele TJ. On the promotion of human flourishing. Proc Natl Acad Sci. 2017 Aug 1;114(31):8148–56.

19. Hanson JA, VanderWeele TJ. The Comprehensive Measure of Meaning: Psychological and Philosophical Foundations. In: Measuring Well-Being [Internet]. Oxford University Press; 2021 [cited 2022 Sep 21]. p. 339–76. Available from: https://academic.oup.com/book/39523/chapter/339351766

20. Young JC, Conover MM, Jonsson Funk M. Measurement Error and Misclassification in Electronic Medical Records: Methods to Mitigate Bias. Curr Epidemiol Rep. 2018 Dec;5(4):343–56.

21. Gianfrancesco MA, Goldstein ND. A narrative review on the validity of electronic health record-based research in epidemiology. BMC Med Res Methodol. 2021 Dec;21(1):234.

22. Getzen E, Ungar L, Mowery D, Jiang X, Long Q. Mining for equitable health: Assessing the impact of missing data in electronic health records. J Biomed Inform. 2023 Mar;139:104269.

23. Liu X, Chubak J, Hubbard RA, Chen Y. SAT: a Surrogate-Assisted Two-wave case boosting sampling method, with application to EHR-based association studies. J Am Med Inform Assoc. 2022 Apr 13;29(5):918–27.

24. Lotspeich SC, Amorim GGC, Shaw PA, Tao R, Shepherd BE. Optimal multiwave validation of secondary use data with outcome and exposure misclassification. Can J Stat. 2023 Mar 31;cjs.11772.

25. Hochberg Y. On the Use of Double Sampling Schemes in Analyzing Categorical Data with Misclassification Errors. J Am Stat Assoc. 1977 Dec;72(360a):914–21.

26. Denny JC, Ritchie MD, Basford MA, Pulley JM, Bastarache L, Brown-Gentry K, et al. PheWAS: demonstrating the feasibility of a phenome-wide scan to discover gene-disease associations. Bioinforma Oxf Engl. 2010 May 1;26(9):1205–10.

27. Denny JC, Bastarache L, Ritchie MD, Carroll RJ, Zink R, Mosley JD, et al. Systematic comparison of phenome-wide association study of electronic medical record data and genome-wide association study data. Nat Biotechnol. 2013 Dec;31(12):1102–11.
